# Supplementary material for: Retinitis Pigmentosa Associated with EYS Gene Mutations: Disease Severity Staging and Central Retina Atrophy
Source: Diagnostics (Basel). 2023 Feb 23;13(5):850. doi: 10.3390/diagnostics13050850 (PMC10000790; doi:10.3390/diagnostics13050850)
Supplement: Supplementary file 1 [file diagnostics-13-00850-s001.zip › diagnostics-2167346-supplementary.pdf]

diagnostics

Table S1. Results of RP-SSS, ERGs and iRORA area of individual patients.

NR	RE SCORE					LE SCORE					IRORA 5 mm		FOCAL ERG		FLICKER 30 Hz ERG	
	BCVA	VF	EZ	Total	Grade	BCVA	VF	EZ	Total	Grade	RE	LE	RE	LE	RE	LE
**1**	4	3	5	**12**	**4**	5	3	5	**13**	**5**	2.2	5.1	NA	NA	0.48	0.71
**2**	5	5	5	**15**	**5**	4	5	5	**14**	**5**	5.3	14.5	0.09	0.14	0.28	0.50
**3**	0	0	1	**1**	**1**	1	0	1	**2**	**1**	0.0	0.2	NA	NA	NA	NA
**4**	5	5	5	**15**	**5**	5	5	5	**15**	**5**	6.9	3.6	NA	NA	0.39	0.35
**5**	4	2	2	**8**	**3**	2	2	5	**9**	**3**	0.0	0.1	0.10	0.15	NA	NA
**6**	0	0	2	**2**	**1**	0	0	2	**2**	**1**	0.0	0.0	0.51	0.70	0.51	0.52
**7**	5	4	4	**13**	**5**	5	4	5	**14**	**5**	0.8	2.8	0.14	0.07	NA	NA
**8**	0	2	4	**6**	**2**	0	1	3	**4**	**2**	2.8	6.4	0.44	0.36	0.48	0.18
**9**	4	0	4	**8**	**3**	4	1	4	**9**	**3**	3.7	5.1	0.36	0.34	1.07	1.04
**10**	3	2	4	**9**	**3**	3	2	4	**9**	**3**	0.0	0.0	NA	NA	0.14	0.44
**11**	5	4	5	**14**	**5**	5	5	5	**15**	**5**	3.7	4.9	NA	NA	0.61	0.50
**12**	4	3	2	**9**	**3**	4	4	2	**10**	**4**	3.7	5.5	NA	NA	0.30	0.26
**13**	4	4	5	**14**	**5**	4	4	5	**14**	**5**	6.1	7.2	NA	NA	0.45	0.53
**14**	1	3	4	**8**	**3**	0	3	3	**6**	**2**	0.8	0.1	NA	NA	1.03	0.44
**15**	5	5	5	**15**	**5**	5	5	NA	**NA**	**NA**	17.6	NA	NA	NA	NA	NA
**16**	3	4	5	**12**	**4**	2	3	2	**7**	**3**	NA	NA	NA	NA	0.52	0.38
**17**	5	5	5	**15**	**5**	5	5	5	**15**	**5**	NA	NA	0.01	0.12	NA	NA

Legend: RP-SSS, retinal pigment stage scoring system; iRORA, incomplete retinal pigment epithelium and outer retinal atrophy; NR, number; RE, right eye; LE, left eye; ERG, electroretinogram; BCVA, best corrected visual acuity; VF, visual field; EZ, ellipsoid zone
